# Chronic traumatic encephalopathy: quantifying risk and clarifying pathogenesis

**DOI:** 10.1007/s00415-020-09734-2

**Published:** 2020-02-06

**Authors:** D. McLauchlan, N. P. Robertson

**Affiliations:** Department of Neurology, Division of Psychological Medicine and Clinical Neuroscience, Cardiff University, University Hospital of Wales, Heath Park, Cardiff, CF14 4XN UK

Chronic traumatic encephalopathy (CTE) was initially described in boxers and colloquially known as ‘punch-drunk syndrome’. However, it has the potential to affect anyone exposed to repeated mild traumatic brain injury and hence represents a significant problem for players and governing bodies of contact sports worldwide. The morbidity of CTE can be significant, and is associated with a higher risk of dementia compared with the normal population. CTE has established pathological criteria from National Institute of Neurological Disorders and Stroke formulated in 2016, with irregular phosphorylated tau deposition in neurons and astroglia around blood vessels found at the base of cortical sulci necessary to confirm the diagnosis.

The syndrome of CTE, and its associated constellation of symptoms are now well described and have been identified in patients and former players engaged in a range of contact sports. The majority of early studies in this field focussed on establishing an understanding of pathophysiogy. However, current challenges for the field lie in quantifying CTE risk from individual contact sports, establishing whether tau is the only contributory pathway in the development of dementia in CTE, establishing novel therapeutic targets and developing a reliable ante-mortem biomarker for diagnosis and prognostication.

This article describes papers that expand our understanding of CTE. The first quantifies risk of neurodegenerative disease and mortality in former players of association football and compares this with the risk of death from other causes. The second study describes the contributions of white matter rarefaction and cerebrovascular pathology to dementia in CTE. Finally the third study describes the performance of a novel PET imaging ligand in predicting symptoms of CTE in traumatic brain injury and distinguishing CTE patients from controls.

## Neurodegenerative disease mortality among former professional soccer players

Association football (soccer) is the most popular sport in the world, and the growing recognition that playing soccer is associated with CTE has significant public health implications. However, the health benefits of playing sport are also recognised to reduce all cause mortality, particularly from cardiovascular causes. A public health policy limiting or restricting access to contact sports, which is not carefully considered, may therefore cause more harm than good. This retrospective case control study identified former professional footballers and compared mortality outcomes with a cohort of matched controls.

7,676 registered soccer players were recruited by searching the records of the Scottish football museum and professional soccer clubs for registered professionals. The records (name and date of birth) were then linked with the community health number (a health record number unique to each individual in Scotland) on a probabilistic basis. Former soccer players were matched to other individuals in the community health record by age, sex and deprivation index in a 1:3 ratio, resulting in a 23,028 strong control cohort. Outcomes were derived from death certificates (all-cause mortality; neurodegenerative disease; circulatory disease and respiratory disease). Prescribing data and deprivation indices were derived from national records. The analysis used cox-proportional hazard models, with a study endpoint of either mortality or December 2016.

Mortality in soccer players was lower until age 70, and increased thereafter (overall hazard ratio 0.87). Mortality from circulatory disease and respiratory disease was lower in soccer players compared with controls, but mortality from neurodegenerative disease was higher and this disparity persisted even when mortality for cardiovascular and respiratory cause was accounted for (hazard ratio 3.53). Players had a higher risk of dementia, motor neurone disease and Parkinson’s disease, and more frequent prescription of dementia related drugs than controls. In a subgroup analysis all-cause mortality and mortality secondary to neurodegenerative disease did not differ between outfield players and goalkeepers, but goalkeepers had fewer prescriptions for dementia related drugs than outfield players, consistent with known lower rates of traumatic brain injury (TBI) in goalkeepers.

*Comment*: Whilst this study is retrospective, the outcomes are based on death certificates and conclusions supported by prescribing records. The method for accounting for deprivation has some limitations as it is based on last known address, potentially missing historical deprivation. Furthermore the algorithm for linking soccer club records to health records is not described in the paper in detail and all the participants were male, limiting generalisability. However, even with these caveats, the evidence that repeated low level concussions increase the risk of neurodegenerative disease and physical activity reduces the risk of cardio-respiratory disease are biologically plausible and supported by multiple outcomes.

Mackay DF et al. NEJM. 2019; 381(19): 1801–8.

## Association of white matter rarefaction, arteriolosclerosis, and Tau with dementia in chronic traumatic encephalopathy

In Alzheimer’s disease, amyloid deposition, neurofibrillary tangles (NFT) and arteriosclerosis all contribute to the development of dementia. CTE is defined by phosphorylated tau deposition around arterioles, but the contribution of other pathological mechanisms to dementia is unknown. Without a clear understanding of the aetiology of dementia in CTE, prospects for therapeutic intervention remain limited. This post-mortem study in a large cohort of former NFL (National Football League—American football) players assesses the contribution of tau and non-tau pathways to the development of dementia in CTE.

This study recruited former NFL players and included all cases meeting pathological criteria for CTE. Neuropathologists assessed the samples blinded to clinical data. Neurofibrillary tangles and tau were measured in the dorso-lateral prefrontal cortex (the area with the highest burden of tau deposition in CTE). Four regions (inferior parietal, middle frontal, superior temporal and occipital) were sampled for white matter rarefaction (WMR: myelin loss, vacuolisation around small blood vessels and reactive astrocyte density) and arteriosclerosis (hyaline thickening of arterioles). Micro bleeds and infarctions were measured throughout the whole brain. Information on symptoms of dementia was made retrospectively by telephone with an interview with a caregiver; dementia diagnosis was according to DSM V criteria. Simultaneous regression modelling assessed contributions to dementia diagnosis.

224 men were recruited; 14 were excluded for incomplete data and 30 were excluded as they died before age 40. 120/180 in the final sample had dementia. Increasing age was associated with higher levels of dementia, WMR and CTE grade (tau deposition). Increasing years of play (a surrogate for number of repetitive TBIs) was associated with increased WMR, higher NFT burden and worse CTE grade. WMR and NFT were equally associated with dementia. Arteriosclerosis was associated with dementia and vascular risk factors, but not years of play. A sensitivity analysis showed that WMR in this cohort was not associated with vascular risk factors or arteriosclerosis.

*Comment*: This study shows that TBI induced axonal damage is a major contributor to dementia in CTE in addition to tau deposition. Although the case ascertainment raises the possibility of selection bias (as the sample was composed of volunteers and entirely male) and the diagnosis of dementia was retrospective and not based on clinical assessment, the findings remain significant. Importantly, the white matter damage in CTE is independent of vascular disease.

Alosco ML et al. JAMA Neurol. 2019;76(11):1298–1308.

## PET-detectable tau pathology correlates with long-term neuropsychiatric outcomes in patients with traumatic brain injury

Currently a diagnosis of CTE can only formally be made post-mortem. The lack of a reliable ante-mortem diagnosis and inability to track disease progression are major limitations in aetiological and therapeutics studies in CTE. This study measured the ability of a novel tau-specific PET ligand to distinguish CTE participants from controls and measure symptoms in the CTE cohort.

The authors recruited participants with severe or chronic repetitive TBI aged over 20 who were free of neurological or psychiatric symptoms prior to TBI, and a cohort of community controls. All participants completed a questionnaire relating to symptoms of CTE (Traumatic encephalopathy syndrome, TES), assessments of cognition (a battery of tasks including MMSE, in addition to tasks focussing on memory, attention and executive function) and psychiatric symptoms (questionnaire measures of apathy, psychosis and depression). All participants underwent quantitative PET with a novel ligand (^11^C-PBB3) shown to reliably detect tau deposition post-mortem in a previous study.

27 participants with TBI (14 with symptoms of CTE) and 15 controls were recruited. The TBI group had higher quantitative binding in the neocortex, particularly in the temporal and occipital regions compared with controls. Higher ^11^C-PBB3 binding was also found in the white matter of the TBI group (frontal, occipital and temporal) compared to controls. The symptomatic TBI group had higher ^11^C-PBB3 binding in the medial frontal white matter compared with the asymptomatic TBI group; grey matter ligand binding did not differ between groups. Medial frontal white matter ^11^C-PBB3 correlated with psychosis scores and localised to superficial white matter.

*Comment*: This PET ligand has been previously shown to reliably localise tau in post-mortem samples; this study shows that quantitative ^11^C-PBB3 PET binding is higher in TBI cases compared with controls, and also in symptomatic compared with asymptomatic patients. Whilst the authors did not correct for multiple comparisons when assessing the correlations between PET and cognitive tasks or psychiatric symptoms, the potential for use as a biomarker is significant. The biological plausibility is strengthened by the pattern of PET binding—showing a frontal and superficial white matter pattern.

Takahata K et al. Brain. 2019; 142: 3265–3279.

